# Overexpression of a cytochrome P450 and a UDP-glycosyltransferase is associated with imidacloprid resistance in the Colorado potato beetle, *Leptinotarsa decemlineata*

**DOI:** 10.1038/s41598-017-01961-4

**Published:** 2017-05-11

**Authors:** Emine Kaplanoglu, Patrick Chapman, Ian M. Scott, Cam Donly

**Affiliations:** 1 Department of Biology, The University of Western Ontario, London, ON N6A 3K7 Canada; 2 London Research and Development Centre, Agriculture and Agri-Food Canada, London, ON N5V 4T3 Canada

## Abstract

Current control of insect pests relies on chemical insecticides, however, insecticide resistance development by pests is a growing concern in pest management. The main mechanisms for insecticide resistance typically involve elevated activity of detoxifying enzymes and xenobiotic transporters that break-down and excrete insecticide molecules. In this study, we investigated the molecular mechanisms of imidacloprid resistance in the Colorado potato beetle, *Leptinotarsa decemlineata* (Say) (Coleoptera: Chrysomelidae), an insect pest notorious for its capacity to develop insecticide resistance rapidly. We compared the transcriptome profiles of imidacloprid-resistant and sensitive beetle strains and identified 102 differentially expressed transcripts encoding detoxifying enzymes and xenobiotic transporters. Of these, 74 were up-regulated and 28 were down-regulated in the resistant strain. We then used RNA interference to knock down the transcript levels of seven up-regulated genes in the resistant beetles. Ingestion of double-stranded RNA successfully knocked down the expression of the genes for three cytochrome P450s (*CYP6BQ15*, *CYP4Q3* and *CYP4Q7*), one ATP binding cassette (ABC) transporter (*ABC-G*), one esterase (*EST1*), and two UDP-glycosyltransferases (*UGT1* and *UGT2*). Further, we demonstrated that silencing of *CYP4Q3* and U*GT2* significantly increased susceptibility of resistant beetles to imidacloprid, indicating that overexpression of these two genes contributes to imidacloprid resistance in this resistant strain.

## Introduction

The Colorado potato beetle (CPB), *Leptinotarsa decemlineata* (Say) (Coleoptera: Chrysomelidae), is a destructive pest of solanaceous crops such as potato, tomato, and eggplant, and its impact on agriculture is measured on a global scale^1^. Left unmanaged, the beetle can completely defoliate plants and cause potato yield losses reaching up to 64 percent^2^. CPB is regarded as one of the most successful insects in its capability to evolve insecticide resistance, having developed resistance to 56 different insecticides since the mid-1950s^3^. Currently, neonicotinoids represent the most commonly used insecticides against this pest due to their effectiveness by seed treatment and systemic movement in the plant^4^. However, persistent use has resulted in selection for neonicotinoid-resistant beetle populations in different areas of the world^5–7^, and this raises great concerns about the sustainability of these insecticides.

Similar to other insecticide-resistant insects, CPB employs several resistance mechanisms to cope with insecticides. These mechanisms include target site insensitivity^8^, decreased penetration^9^, increased excretion^10^, and metabolic detoxification of insecticides^11^. Of these, metabolic resistance is the best understood mechanism, and is believed to be derived from an ancestral ability to neutralize dietary toxins^12^. In fact, the success of CPB in overcoming insecticides is in part attributed to the fact that it has coevolved with its host plants in the family Solanaceae^13^ which produce extremely toxic compounds known as glycoalkaloids^14^. Hence, having an enhanced ability to detoxify plant toxins is thought to enable the beetle to rapidly develop resistance to other toxins, including insecticides.

Metabolic resistance is caused by increased break-down and excretion of insecticide molecules, and is achieved by elevated activity of detoxifying enzymes^15^ and xenobiotic transporters^16^ in the insecticide-resistant insects. The most important detoxifying enzymes and xenobiotic transporters involved in metabolic resistance are cytochrome P450s (CYPs) and esterases (ESTs) in phase I direct metabolism^15, 17^, glutathione S-transferases (GSTs) and UDP-glycosyltransferases (UGTs)^18–20^ in phase II conjugation, and ATP-binding cassette (ABC) transporters in phase III excretion^16^.

An important feature of metabolic resistance to insecticides is the transcriptional up-regulation of detoxifying enzyme and ABC transporter genes in insecticide-resistant insects, which usually results in constitutive overexpression of the aforementioned proteins^21^. In fact, constitutive overexpression of these genes is arguably the most common mechanism resulting in resistance seen in many insecticide-resistant insects^15^. For instance, constitutive overexpression of multiple CYPs, ESTs, GSTs, UGTs, and ABC transporters is associated with neonicotinoid resistance in the whitefly, *Bemisia tabaci* ^22^, in the tarnished plant bug, *Lygus lineolaris* ^23^, and in the cotton aphid, *Aphis gossypii* ^24^ as well as pyrethroid resistance in the house fly, *Musca domestica* ^25^.

In CPB, metabolic resistance is the mechanism involved in resistance to carbamate^10^, pyrethroid^26^, organophosphate^11^, and abamectin^27^ classes of insecticides. Further, there is some evidence that metabolic resistance is also responsible for neonicotinoid resistance as well^28, 29^. However, support for the role of metabolic resistance to many insecticides mainly comes from studies using insecticide synergists that inhibit the activity of the detoxifying enzymes *in vivo* and increase the potency of the insecticides. For example, using piperonyl butoxide (PBO), and S,S,S-tributylphosphorotrithioate (DEF), which inhibit CYP and EST enzymes, respectively, it was shown that resistance to imidacloprid, the most recognized first-generation neonicotinoid, can be reduced significantly in CPB^28, 29^. Although these early studies provided the first evidence that metabolic resistance played a role in imidacloprid resistance in CPB, they failed to identify specific genes involved in the process. Furthermore, recent studies demonstrated that several genes encoding CYPs, ESTs, GSTs, and ABC transporters are overexpressed constitutively or upon imidacloprid exposure in imidacloprid-resistant CPB populations compared to sensitive populations^30, 31^. Also, Clements *et al*. identified a specific CYP gene (*CYP9Z26*) whose constitutive overexpression was associated with imidacloprid resistance in a CPB population from the Central Sands region of Wisconsin^32^, providing further evidence for the role of metabolic detoxification in imidacloprid resistance.

Despite considerable evidence that imidacloprid resistance in CPB is conferred by metabolic mechanisms, there is still limited information about which genes are involved. Thus, the main goal of this study was to further our knowledge on the genes involved in imidacloprid resistance in CPB. To accomplish this, we used RNA sequencing (RNA-seq) to identify genes encoding detoxifying enzymes and ABC transporters with constitutively increased transcription levels in an imidacloprid-resistant strain of CPB compared to a sensitive strain. We then used RNA interference (RNAi) to knock-down the expression of selected genes in the resistant beetles and evaluated the phenotypic effects of silencing resistance-related genes on those imidacloprid resistance mechanisms. Our results confirmed that constitutive overexpression of detoxifying enzymes plays a role in resistance to imidacloprid in CPB.

## Results

### Identification of differentially expressed genes between resistant and sensitive beetles

The mRNA expression profiles of imidacloprid-resistant (RS) and imidacloprid-susceptible (SS) CPB were compared to identify constitutively differentially expressed genes between the two strains. Sequencing of transcripts from the two strains yielded a total of 351,002,722 reads (Supplementary Table S1). Of these, a total of 65,918,757 and 64,399,443 high-quality 100 bp reads from the RS and SS CPB, respectively, uniquely mapped to the previously published reference transcriptome^33^, and were used for differentially expressed sequence (DESeq) analysis^34^. A total of 7572 differentially expressed contigs were identified between the two strains based on a false discovery rate (FDR)-corrected significance value of 0.001 (Supplementary Fig. S1). Of these, 4220 were up-regulated and 3352 were down-regulated in the RS. Differentially expressed contigs were then manually screened to identify transcripts encoding detoxifying enzyme and ABC transporter genes. A total of 102 contigs corresponding to genes of interest were identified; of these, 74 showed increased, while 28 showed decreased, transcript levels in the RS (Supplementary Table S2). Of the 74 up-regulated contigs, 24 corresponded to CYP, 13 to UGT, 14 to EST, 15 to GST, and 8 to ABC transporter encoding transcripts. Among the 28 down-regulated contigs, there were 6 CYP, 2 UGT, 9 EST, 4 GST, and 7 ABC transporter encoding transcripts.

### Validation of differentially expressed sequences

**Figure 1:**
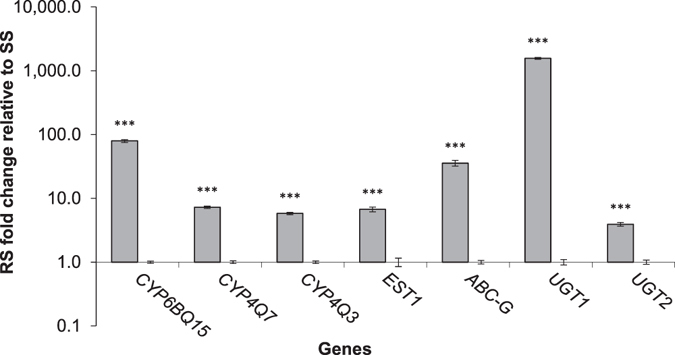
qPCR validation of DESeq analysis on independent biological samples. Fold increase in normalized mRNA expression levels of seven genes in the RS beetles relative to normalized expression levels in the SS beetles (set to one). Data are expressed as mean relative quantity ± SEM. Asterisks represent significant changes in the mRNA transcript levels in *t*-tests (***P ≤ 0.001), n = 3.

Differential expression of genes was validated by quantitative PCR (qPCR) on independent biological samples. Seven genes, including three putative CYPs (*CYP6BQ15*, *CYP4Q3*, and *CYP4Q7*), one putative ABC transporter in the G subfamily (here referred to as *ABC-G*), one putative EST (here referred to as *EST1*), and two putative UGTs (here referred to as *UGT1*, and *UGT2*) were selected based on their fold change differences in DESeq analysis. Some sequences with high fold increases were not tested because efficient qPCR primers could not be found in the annotated sequences. DESeq results showed that the gene annotated as *CYP4Q7* was down-regulated in the RS, but the qPCR results showed an opposite trend for this gene (Fig. 1 and Supplementary Table S3). This disagreement of the RNA-seq results with the qPCR confirmation may have been caused by poor alignment of sequencing reads to the reference transcriptome in the RNA-seq analysis. All seven genes chosen for analysis were significantly up-regulated in the RS compared to the SS based on qPCR results (Fig. 1). Among all genes analyzed in the RS compared to SS based on qPCR results, the order of highest to lowest fold increase was *UGT1* (~1565 fold) > *CYP6BQ15* (~80 fold) > *ABC-G* (~36 fold) > *CYP4Q7* (~7 fold) > *EST1* (~6 fold) > *CYP4Q3* (~5.8 fold) > *UGT2* (~4 fold). All seven up-regulated genes were selected for further investigation.

### RNAi of selected genes

**Figure 2:**
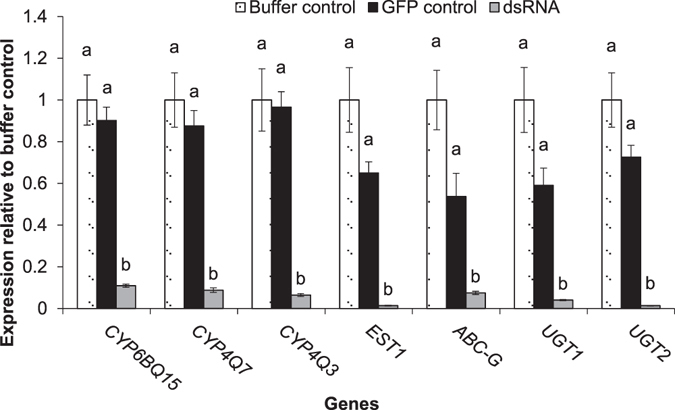
Confirmation of RNAi knock-down of seven genes in the RS beetles through qPCR analysis. Normalised expression of seven target genes in the RS beetles that ingested either *E. coli* HT115 producing dsRNA for seven genes, *E. coli* producing dsRNA for *GFP*, or buffer. Data are expressed as mean relative quantity ± SEM, n = 3. Letters placed above bars denote significant differences in mRNA levels for each gene. Means with the same letter are not significantly different (*P* > 0.05) according to Tukey’s HSD test (one-way ANOVA).

To further investigate their functions in imidacloprid resistance, we employed RNAi and knocked down the expression of *CYP6BQ15*, *CYP4Q3*, *CYP4Q7*, *ABC-G*, *EST1*, *UGT1*, and *UGT2* in the RS CPB. RNAi of target genes was accomplished by producing double-stranded RNA (dsRNA) specific for the selected genes in bacteria (Supplementary Fig. S2 and Table S4) and feeding the bacteria to RS adults. Silencing of the genes was confirmed using qPCR. Relative transcript levels of the seven genes were analyzed in the beetles that fed on potato leaves treated with buffer (buffer control), dsRNA for green fluorescent protein (*GFP*) (GFP control), or dsRNA for the target genes for four days. Results from one-way ANOVA tests showed that feeding on dsRNA for the target genes resulted in a significant reduction in the mRNA levels of all genes (*CYP6BQ15* (*F*_2,6_ = 16.7, *P* = 0.0035), *CYP4Q7* (*F*_2,6_ = 10.9, *P* *=* 0.010), *CYP4Q3* (*F*_2,6_ = 19.21, *P* = 0.0024), *EST1* (*F*_2,6_ = 120.5, *P* = 1.43E-05), *ABC-G*, (*F*_2,6_ = 18.03, *P* = 0.0029), *UGT1* (*F*_2,6_ = 26.4, *P* = 0.0010), and *UGT2* (*F*_2,6_ = 102.8, *P* = 2.28E-05)). The Tukey’s honest significant difference (HSD) test showed that mRNA levels of the seven genes did not differ significantly in beetles fed on dsRNA-*GFP* and buffer treated potato leaves (Fig. 2).

### Phenotypic effects of silencing genes on imidacloprid resistance

**Figure 3:**
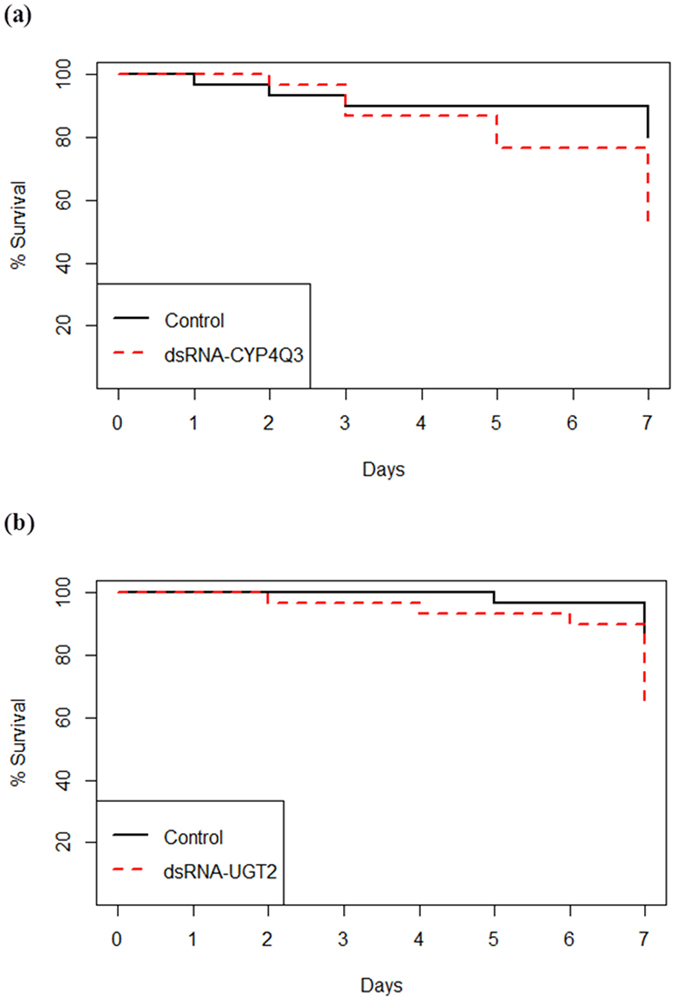
Kaplan-Meier survival curves illustrating the percent survival of the RS beetles exposed to LD_20_ of imidacloprid. Beetles either ingested *E. coli* HT115 (control) or *E. coli* HT115 producing dsRNA for **(a)** *CYP4Q3*, or **(b)** *UGT2*. Ingestion of dsRNA significantly increased the sensitivity of the RS beetles to imidacloprid after the two genes were silenced individually according to Log-rank tests (*P* < 0.05), n = 30.

To determine whether silencing of *CYP6BQ15*, *CYP4Q3*, *CYP4Q7*, *ABC-G*, *EST1*, *UGT1*, or *UGT2* genes would increase the susceptibility of the RS to imidacloprid, seven-day bioassays were performed with RS CPB. The beetles were fed with potato leaves treated with untransformed bacteria (control) or with bacteria producing dsRNA for the target genes for four days prior to imidacloprid exposure. The percent survival of beetles exposed to the LD_20_ of imidacloprid was analyzed using Kaplan-Meier survival analysis and Log-rank tests which determined that silencing *CYP4Q3* and *UGT2* significantly increased the toxicity of imidacloprid in the RS CPB. Knocking down the mRNA levels of *CYP4Q3* and *UGT2* increased the beetle mortality significantly upon exposure to LD_20_ of imidacloprid (log rank χ² = 4.3, df = 1, *P* = 0.037, n = 30 for *CYP4Q3* and log rank χ² = 4.3, df = 1, *P* = 0.038, n = 30 for *UGT2*) (Fig. 3A and B). Overall, the average mortality of the RS CPB increased from 20.0% to 46.7% and 13.3% to 36.7% compared with the control when beetles were fed with dsRNA-*CYP4Q3* and dsRNA-*UGT2*, respectively. Silencing of *EST1* and *CYP4Q7* increased mortality from 20.0% to 33.3% and from 20.0% to 30.0%, respectively, but the increases were not statistically significant (*P* > 0.05) (Supplementary Fig. S3). Similarly, feeding RS with dsRNA-*GFP*, dsRNA-*UGT1*, dsRNA-*ABC-G*, or dsRNA-*CYP6BQ15* did not result in a significant increase in the toxicity of imidacloprid (*P* > 0.05) (Supplementary Fig. S3).

### Double-knockdown of *CYP4Q3* and *UGT2* genes

**Figure 4:**
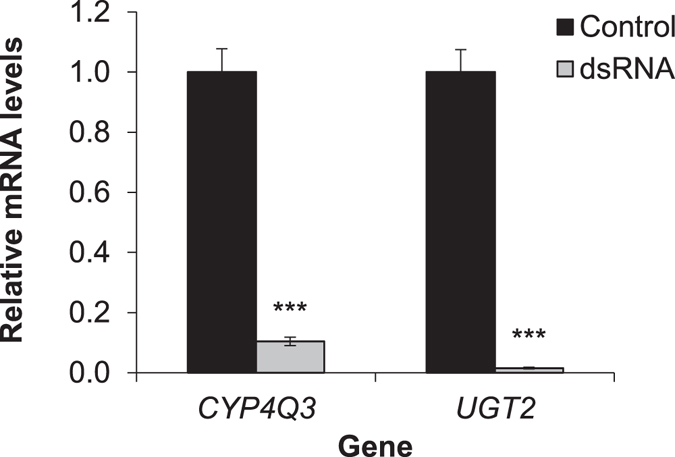
qPCR confirmation of simultaneous RNAi knock-down of *CYP4Q3* and *UGT2*. The RS beetles ingested *E. coli* HT115 or a 1:1 ratio of two *E. coli* HT115 strains producing dsRNA for the two target genes. Normalized mRNA quantities are set to one in the control and the change in mRNA levels in the dsRNA fed beetles was calculated relative to the control. Data are expressed as mean relative quantity ± SEM. Asterisks represent significant changes in *t*-tests (***P ≤ 0.001), n = 3.

**Figure 5:**
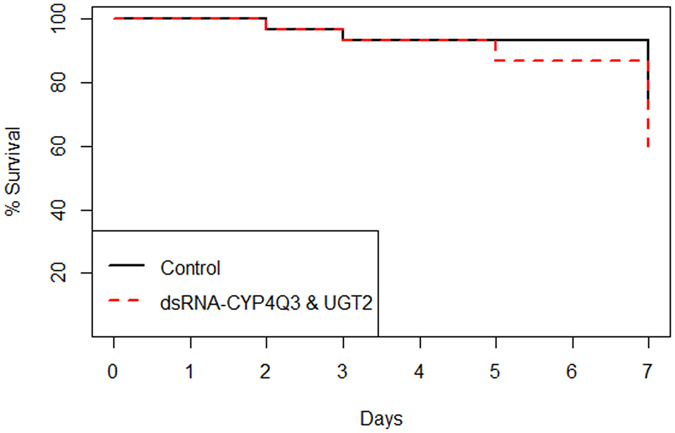
Kaplan-Meier survival curve illustrating the percent survival of the RS beetles exposed to LD_20_ of imidacloprid after simultaneous silencing of *CYP4Q3* and *UGT2*. The beetles either ingested *E. coli* HT115 or 1:1 ratio of two *E. coli* HT115 strains producing dsRNA for *CYP4Q3* and *UGT2*. RNAi of the two genes did not increase the sensitivity of the RS beetles to imidacloprid after being silenced simultaneously, according to Log-rank tests (*P* > 0.05), n = 30.

To test for a possible synergistic action on imidacloprid resistance, transcript levels of *CYP4Q3* and *UGT2* were knocked down simultaneously through RNAi. These two genes were selected based on the fact that their individual knock-down in the RS beetles significantly increased the toxicity of imidacloprid. qPCR results confirmed that mRNA transcript levels of *CYP4Q3* and *UGT2* were knocked down significantly (independent samples *t*-tests, *t*_(4)_ *=* 8.01, and *P* = 0.00065 for *CYP4Q3* and *t*_(4)_ = 10.68 and *P* = 0.00021 for *UGT2*) when the beetles were fed with a 1:1 mixture of two bacterial strains producing dsRNA for the two genes (Fig. 4). Simultaneous silencing of the two genes increased the mortality of the RS beetles from 26.7% to 40.0% upon exposure to LD_20_ of imidacloprid, however, the increase was not statistically significant (*P* > 0.05) (Fig. 5).

## Discussion

In this study, we compared the mRNA expression profiles of an imidacloprid-resistant and an imidacloprid-sensitive strain of CPB to uncover the molecular basis for imidacloprid resistance. Our results revealed that the imidacloprid-resistant strain of CPB constitutively overexpresses multiple genes encoding CYPs, ESTs, GSTs, UGTs, and ABC transporters, relative to the sensitive strain. We also investigated the potential contribution of seven upregulated genes in imidacloprid resistance through RNAi, and identified two detoxifying enzyme genes, a CYP, *CYP4Q3*, and a UGT, *UGT2*, whose overexpression contributes to imidacloprid resistance. We showed that RNAi knock - down of transcription for *CYP4Q3* and *UGT2* genes resulted in a significant increase in susceptibility of resistant beetles to imidacloprid. Hence, based on our results, we conclude that metabolic resistance plays a significant role in imidacloprid resistance in CPB, and there are several genes involved in the process.

Among these five protein superfamilies, we found that the highest number of detoxifying enzyme transcripts upregulated in the RS corresponded to CYP enzymes. CYPs comprise a superfamily of metabolic enzymes present in all kingdoms of life^17^, and there is considerable evidence for their involvement in insecticide resistance. These enzymes are highly diverse and capable of conferring resistance to all classes of insecticides^15^. For instance, through RNAi studies, several studies have shown that constitutive upregulation of CYPs mediates resistance to pyrethroid insecticides in the diamondback moth, *Plutella xylostella* ^35^, the carmine spider mite, *Tetranychus cinnabarinus* ^36^, and the red flour beetle, *Tribolium castaneum* ^37^. Additionally, CYPs are involved in resistance to neonicotinoids in the small brown plant hopper, *Laodelphax striatellus* ^38^ and the tobacco whitefly, *B. tabaci* ^39^, to organophosphates in the tobacco cutworm, *Spodoptera litura* ^40^, and to abamectin in *P*. *xylostella* ^41^. Furthermore, a recent study by Clements *et al*. analyzed the transcriptomes of two imidacloprid-resistant populations of CPB from the Central Sands region of Wisconsin and showed that a CYP enzyme, *CYP9Z26*, was constitutively overexpressed in one of the resistant populations^31^. Further studies showed that RNAi of *CYP9Z26* results in reduction of imidacloprid resistance^32^. In our study, *CYP9Z26* was not overexpressed in our RS strain based on our RNA-seq data, which implies that different CYP genes may be up-regulated in different resistant populations. However, here we show that RNAi of another CYP gene, *CYP4Q3*, also significantly reduces imidacloprid resistance in CPB. Therefore, our finding provides further evidence for the role of CYP enzymes in imidacloprid resistance and the *CYP4Q3* gene represents another example from the CYP superfamily shown to contribute to imidacloprid resistance in this beetle.

In addition to *CYP4Q3*, we identified a putative UGT enzyme encoding transcript, here referred to as *UGT2*, that is also involved in imidacloprid resistance in CPB. Similar to CYPs, UGTs also form a superfamily of detoxifying enzymes and are found in all living organisms^42^. These enzymes catalyze the conjugation of sugar molecules to a broad range of substrates including plant secondary metabolites. Such reactions increase the solubility of toxic compounds^20^ and facilitate their excretion from the body, allowing insects to thrive on toxin laden diets^43^. In vertebrates, UGT enzymes are well studied, and are considered to be one of the most important drug metabolizing enzymes. It is thought that UGTs, alongside CYPs, are responsible for detoxifying the majority of clinical drugs in humans^44^. However, until recently, the potential contribution of UGTs to insecticide resistance has mainly gone unaddressed, and the only evidence for their involvement comes from studies showing that transcript levels of UGTs are upregulated constitutively or upon insecticide exposure in resistant insects^22, 45^. In this study, for the first time, we have shown that RNAi of a UGT gene increases toxicity of imidacloprid in CPB. Further, this is also the first study to infer a role for a UGT enzyme in imidacloprid resistance in insects.

Although RNAi of *CYP4Q3* and *UGT2* individually resulted in significant increases in beetle mortality upon imidacloprid exposure, this only accounted for a fraction of the resistance exhibited by the RS beetles. However, we found no evidence of synergistic action by the two genes, as simultaneous silencing of *CYP4Q3* and *UGT2* did not increase the mortality of the beetles significantly after imidacloprid exposure (Fig. 5). A similar lack of phenotype, or reduction in phenotype when simultaneously silencing two genes, has also been observed in previous studies. For instance, simultaneous silencing of two essential genes, β-actin (*ACT*) and Shrub (*SHR*), resulted in reduced mortality of CPB larvae compared with the mortality when the two genes were silenced individually^46^. In addition, studies conducted in the red flour beetle, *T. castaneum*, and the cotton bollworm, *Helicoverpa armigera*, found no indication of synergism when multiple genes were targeted simultaneously^47, 48^. Although these studies did not target insecticide resistance-related genes, they imply that targeting two genes simultaneously does not necessarily result in a more sensitive phenotype. However, different gene combinations may give different results as simultaneous silencing of six CYP genes in *T*. *cinnabarinus* was shown to have a greater effect on pyrethroid resistance than silencing them individually^36^.

Because some CYP enzymes have broad substrate specificity, they can metabolize a wide range of compounds, including insecticides, with similar modes of action, often resulting in cross resistance^49^. For instance, an imidacloprid - resistant population of CPB was shown to have cross resistance to several other neonicotinoid insecticides, including clothianidin, acetamiprid, thiacloprid and thiamethoxam, despite the fact that the population had never been exposed to these insecticides^28^. However, it is unknown whether the same genes are responsible for this cross resistance as multiple detoxifying enzymes have been shown to be upregulated in imidacloprid- resistant beetles, both in our study and also in other previously analysed populations^30, 31^. In *B. tabaci*, constitutive overexpression of a specific CYP gene, *CYP6CM1*, is associated with resistance to two neonicotinoid insecticides, imidacloprid and thiamethoxam^50, 51^; yet, *in vitro* functional studies have shown that recombinant *CYP6CM1vQ* enzyme can metabolise several neonicotinoids including imidacloprid, but not thiamethoxam^52, 53^. Although we did not examine whether *CYP4Q3* and *UGT2* are involved in cross resistance to other neonicotinoids in this study, such experiments could be the basis for future studies.

Our results for the remaining five genes tested, *CYP6BQ15*, *CYP4Q7*, *EST1*, *ABC-G*, and *UGT1*, implied that silencing of these genes does not affect the toxicity of imidacloprid significantly in CPB. We observed only a slight increase in the mortality of the beetles when *EST1* and *CYP4Q7* were knocked down, 13.3% and 10.0%, respectively, and silencing of *CYP6BQ15*, *ABC-G*, and *UGT1* had no effect on the imidacloprid resistance. However, we cannot completely rule out their contribution to resistance because the mRNA levels of these genes had the highest fold increases in the resistant beetles compared to sensitive beetles. Therefore, it is possible that RNAi failed to reduce the mRNA levels enough to cause a significant reduction in protein levels, hence, yielding no altered effects to imidacloprid toxicity. However, it is also important to note that mRNA levels do not always accurately reflect functional protein levels^54^. Thus, the transcriptomic data need to be complemented with proteomic data to investigate the correlation between mRNA and protein levels to rule out any role for these genes in resistance.

In conclusion, our results provide evidence for metabolic resistance as the mechanism for imidacloprid resistance in the RS CPB. The most important finding of this work was the identification of two detoxifying enzymes that play roles in imidacloprid resistance. The constitutive overexpression of these genes presumably allows resistant beetles to metabolize insecticide molecules more efficiently, resulting in enhanced resistance. Our results also imply that imidacloprid resistance in the beetle is controlled by multiple genes, some of which still remain to be identified. The knowledge gained from this study is important as it gives us new opportunities to develop novel pest control strategies that can exploit the mechanisms mediating resistance. For instance, RNAi knock-down of resistance-related genes, in combination with chemical insecticides, can offer a new pest control strategy. This could significantly reduce chemical insecticide use and lessen the possibility of resistance development by the target pests.

## Materials and Methods

### CPB strains

Beetles were maintained at the London Research and Development Centre (LRDC), Agriculture and Agri-Food Canada (AAFC), London, ON, Canada. The imidacloprid-susceptible strain, SS, was originally collected in 1991 from the LRDC research farm, and has been in continuous culture over 160 generations without pesticide exposure. The imidacloprid-resistant strain, RS, was originally collected in 1997 from a potato field in Long Island, NY, USA, and was maintained for 51 generations under selection for imidacloprid-resistance at the Department of Entomology, Michigan State University, East Lansing, MI, USA^55^. The strain was obtained by AAFC in 2013 and has been reared for more than 10 generations without insecticide exposure. The beetles were maintained on potato plants (*Solanum tuberosum* var. Kennebec) at 25 °C, 50% relative humidity, and 16:8 h light:dark photoperiod. For all experiments, one to three day-old mixed-sex adult beetles were used. The resistance ratio (RR) of RS compared to the SS was calculated from LD_50_ of imidacloprid for both strains (Supplementary Table S5). The RR was 25.3 as determined by topical application bioassays^29^.

### Chemicals

Technical grade imidacloprid (99.4%) was provided by Bayer CropScience Canada, Inc. (Guelph, ON) and was dissolved in analytical grade acetone for topical applications.

### Total RNA extraction and mRNA sequencing

The SS and RS CPB were dissected to harvest midgut, fat body, and Malpighian tubules. These tissues were selected because expression of detoxifying enzymes and ABC transporters are often enriched in these tissues^19, 30, 56, 57^. The three tissues were pooled and total RNA was isolated using an RNeasy Plant Mini Kit (Qiagen). Three biological replicates per strain were done and each biological replicate consisted of a pool of total RNA extracted from five beetles. The quality and quantity of the total RNA samples were assessed using a 2100 Bioanalyzer (Agilent) and a Qubit RNA HS (High Sensitivity) Assay kit (Thermo Fisher Scientific), respectively. cDNA library construction, using the Illumina TruSeq stranded mRNA library prep kit, and sequencing of cDNA libraries on the Illumina HiSeq 2000 platform were performed at the McGill University and Génome Québec Innovation Centre (Montreal, QC, Canada) following the 100 bp single-end reads protocol (Illumina). Adapter sequences were trimmed using Scythe adapter trimmer (https://github.com/vsbuffalo/scythe) and the reads having <20 nt after trimming were discarded. mRNA-seq reads were mapped to the previously published reference CPB transcriptome^33^ using the Burrows-Wheeler Alignment (q = 30) tool^58^. Uniquely mapping reads were imported to statistical computing language R^59^ and the DESeq package version 1.18.0^34^ was used to identify differentially expressed sequences between the two strains. Differentially expressed contigs had an absolute value of log_2_Fold change of ≥1 and an adjusted *P*-value (*P*adj) of ≤0.001 after the Benjamini-Hochberg FDR^60^. Finally, the differentially expressed contigs were manually screened to find the ones encoding detoxifying enzymes and ABC transporters.

### cDNA synthesis and validation of DESeq results using qPCR

To validate DESeq results, total RNA was extracted from independent biological samples as described above. Total RNA was treated with Turbo DNA-free DNase (Ambion) to eliminate contaminating genomic DNA and the quality and quantity of total RNA was assessed using the 2100 Bioanalyzer (Agilent). cDNA was synthesized from 1 µg of total RNA per 20 µL reaction volume using a Superscript III First-Strand Supermix Kit (Invitrogen). qPCR reactions were performed using a SensiFAST SYBR No-ROX Mix Kit (Bioline), forward and reverse primers at 500 nM each and 2.5 µL of a 1:2 dilution of cDNA template in 10 µL reactions. A two-step cycling profile (95 °C for 2 min for one cycle, and 95 °C for 5 s and 60 °C for 30 s for 40 cycles) was used for quantitation of target genes using a CFX96 Real-Time Detection System (Bio-Rad). Melt-curve analysis was performed for each qPCR run to confirm amplification of a single product, and no-template controls were included to ensure reagents were free of contaminants. Three biological replicates were done from each strain and all samples were run in technical triplicate. Transcript abundance of target genes was normalized to the geometric mean of three endogenously expressed reference genes: ribosomal protein (*L8E*), ADP-ribosylation factor 1 (*ARF1*), and translation elongation factor 1α (*EF1α*)^61, 62^. The relative abundance of transcript difference for target genes was estimated in the RS beetles and the SS beetles using the 2^−ΔΔCt^ method^63^. *T*-tests were performed using R^59^ to determine statistical significance of changes in the expression levels of the target genes. All qPCR primers were validated^64^ to comply with minimum information for publication of quantitative real-time PCR experiment guidelines^65^ (Supplementary Table S6).

### Double-stranded RNA (dsRNA) production

dsRNA was produced in *Escherichia coli* HT115 (DE3) using RNAi vector L4440^66^. The vector was a gift of Andrew Fire (available from Addgene, plasmid # 1654). Gene specific primers were designed to have restriction enzyme cut sites to amplify 384–420 bp PCR products from target genes. All primer pairs contained a *Not*I cut site on the forward primer (5′-GCGGCCGC-3′) and a *Sal*I cut site on the reverse primer (5′-GTCGAC-3′), at the 5′ end (Supplementary Table S7). cDNA from RS beetles was used as a template for PCR. Double digestions of L4440 plasmid and purified PCR products were performed with restriction enzymes *Not*I and *Sal*I (New England Biolabs). Digested PCR-generated fragments were ligated into linearized plasmid using the T4 DNA Ligase Kit (Invitrogen). HT115 cells were transformed with ligation reactions and positive colonies were identified using colony PCR. Cloning of the correct sequences into L4440 was confirmed through sequencing of plasmid preparations (Supplementary Table S8). To produce dsRNA, HT115 cells were grown in Luria Bertani medium containing 100 μg mL^−1^ ampicillin and 12.5 μg mL^−1^ tetracycline until OD_600_ was ~0.4–0.6. Cells were induced to produce dsRNA by adding 1 mM isopropyl-β-D-thiogalactopyranoside and incubating cells for an additional 6 h at 37 °C with shaking. The culture was centrifuged at 10,000 g for 10 min at 4 °C and pellet was washed once with 1× phosphate buffered saline (PBS). The pellet was re-suspended in 1× PBS buffer to concentrate the culture 10×. To confirm dsRNA production in HT115, total nucleic acid was extracted from 1 mL culture using a MasterPure Complete DNA and RNA Purification Kit (Illumina) and was analyzed by electrophoresis through 1.5% agarose gels (Supplementary Fig. S2). dsRNA for a *GFP* gene was also produced as a negative control. The template for *GFP* was GFP::L4440 plasmid that contains the full-length *GFP* gene sequence (available from Addgene, plasmid # 11335).

### dsRNA feeding to silence target genes

HT115 cells producing dsRNA were fed to the RS beetles using the protocol described^67^, with minor modifications. Potato leaves were dipped into the HT115 suspensions, containing dsRNA for the target genes or *GFP*, or into 1× PBS buffer. The leaves were dried under airflow on a metal mesh for 1 h and one leaf was placed per Petri dish (50 × 9 mm) lined with moist filter paper (Whatman qualitative no. 5). RS beetles were starved for 2 h and one beetle was placed per Petri dish. The beetles were allowed to feed *ad libitum* on the treated leaves for four days. After four days, the beetles were dissected and midgut, Malpighian tubule, and fat body tissues were harvested. The three tissues were pooled and RNA extraction, cDNA synthesis, and qPCR reactions were performed as described previously to confirm silencing of genes in the RS beetles. The mRNA transcript levels of target genes were analyzed using one-way ANOVA followed by Tukey’s HSD *post hoc* analysis in R^59^. To silence two genes simultaneously, two strains of HT115 producing dsRNA for each gene were grown separately and then mixed at a 1:1 ratio prior to feeding.

### Bioassays

The RS beetles were fed potato leaves dipped in suspensions of HT115 or in HT115 producing dsRNA for *GFP* or for the target genes. As before, the beetles fed on the treated leaves *ad libitum* for four days. After four days, beetles were topically exposed to 2.7 μg beetle^−1^ of imidacloprid (LD_20_ for RS beetles) as described^5^. The beetles were provided with fresh treated leaves daily after imidacloprid exposure, and survivorship was monitored daily for seven days. Thirty beetles were used in each condition. The beetles that were moribund and dead were counted at the end of seven days using the criteria described^29^. Kaplan-Meier survival analysis and Log-rank tests were performed using R^59^ to determine whether differences existed in survival between the control and treatment groups.

## Electronic supplementary material

Supplementary Information
