# Supplementary material for: Overexpression of a cytochrome P450 and a UDP-glycosyltransferase is associated with imidacloprid resistance in the Colorado potato beetle, Leptinotarsa decemlineata
Source: Sci Rep. 2017 May 11;7:1762. doi: 10.1038/s41598-017-01961-4 (PMC5431904; doi:10.1038/s41598-017-01961-4)
Supplement: Supplementary file 1 — Supplementary Information [file 41598_2017_1961_MOESM1_ESM.pdf]

**Supplementary Material**

**Overexpression of a cytochrome P450 and a UDP-glycosyltransferase is associated with imidacloprid resistance in the Colorado potato beetle, *Leptinotarsa decemlineata***

Emine Kaplanoglu[1,2], Patrick Chapman[2], Ian M. Scott[1,2] and Cam Donly[1,2,]*

[1]Department of Biology, The University of Western Ontario, London, ON, N6A 3K7, Canada

[2]London Research and Development Centre, Agriculture and Agri-Food Canada, London, ON, N5V 4T3, Canada

*Author for correspondence: Cam Donly

E-mail: cam.donly@agr.gc.ca

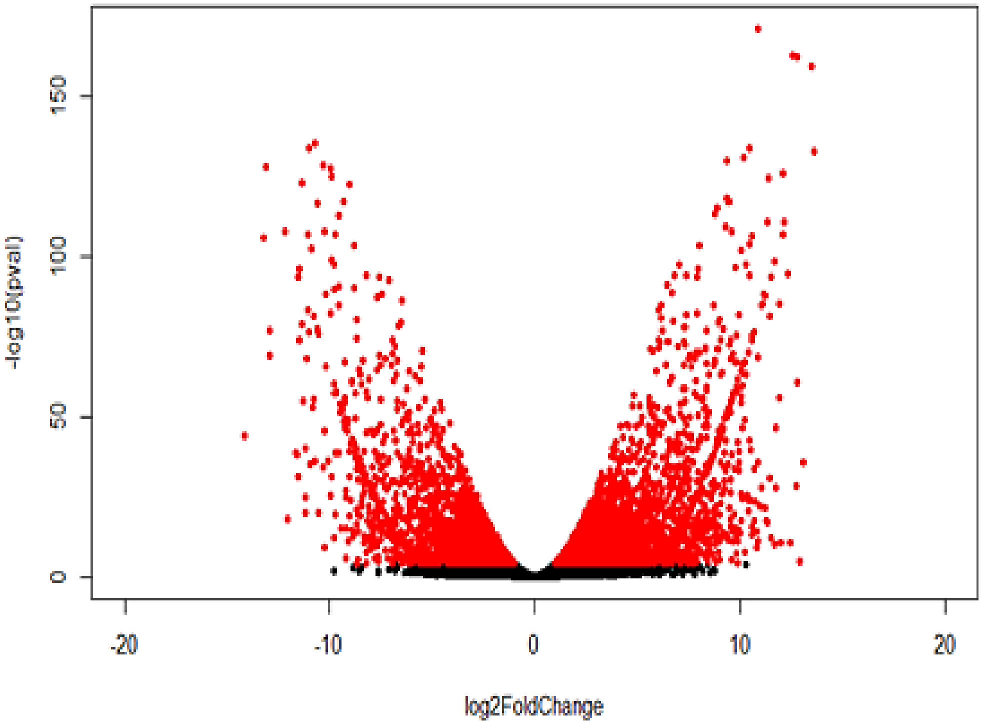


**Figure S1. Volcano plot showing differentially expressed contigs between the RS and SS strains of the Colorado potato beetle.** 7572 contigs showed differential expression, and of these 4220 showed increased and 3352 showed decreased transcript levels in the RS beetles. Contigs that were differentially expressed at FDR of ≤ 0.001 and fold change of $|\log2| \geq 1$ are colored red.

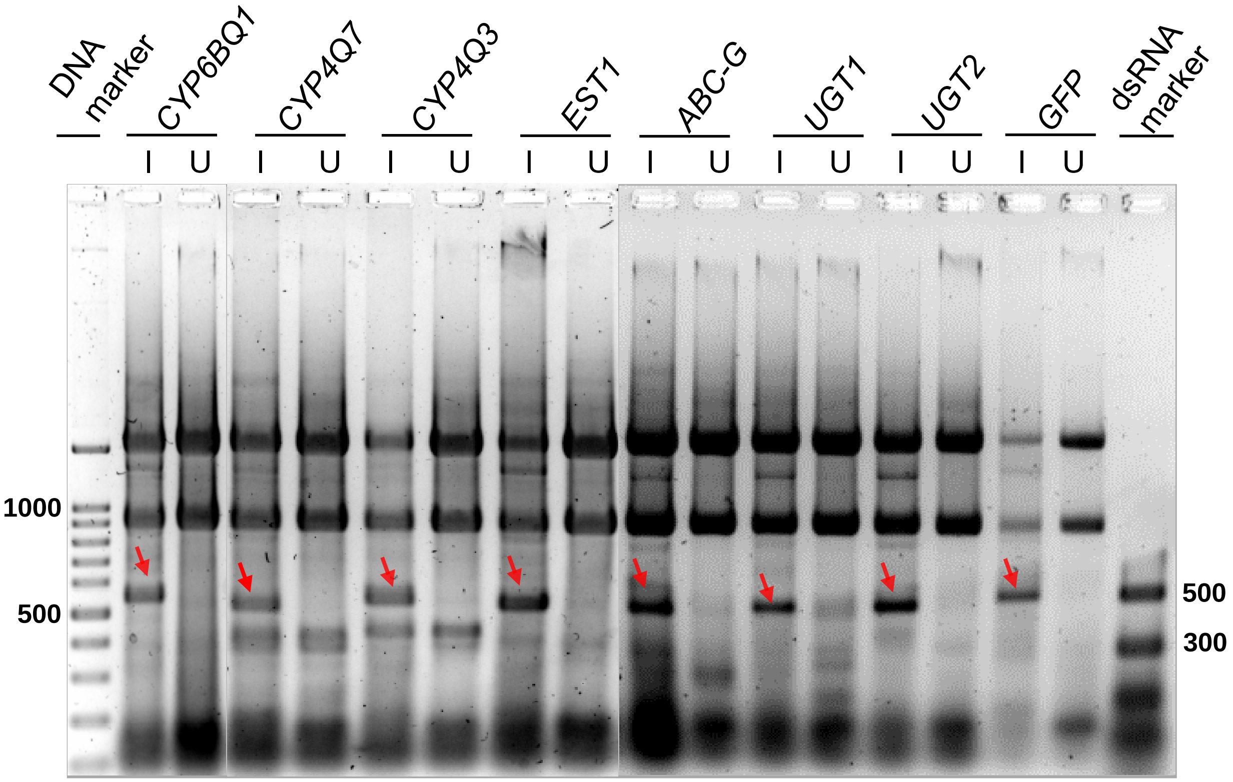


**Figure S2. Production of dsRNA in *E. coli* HT115 for target genes.** I and U indicate lanes loaded with a total RNA sample extracted from bacteria that were induced or not induced with IPTG, respectively. The positions of dsRNA species are marked with red arrows. Sizes of nucleic acid markers are as indicated.

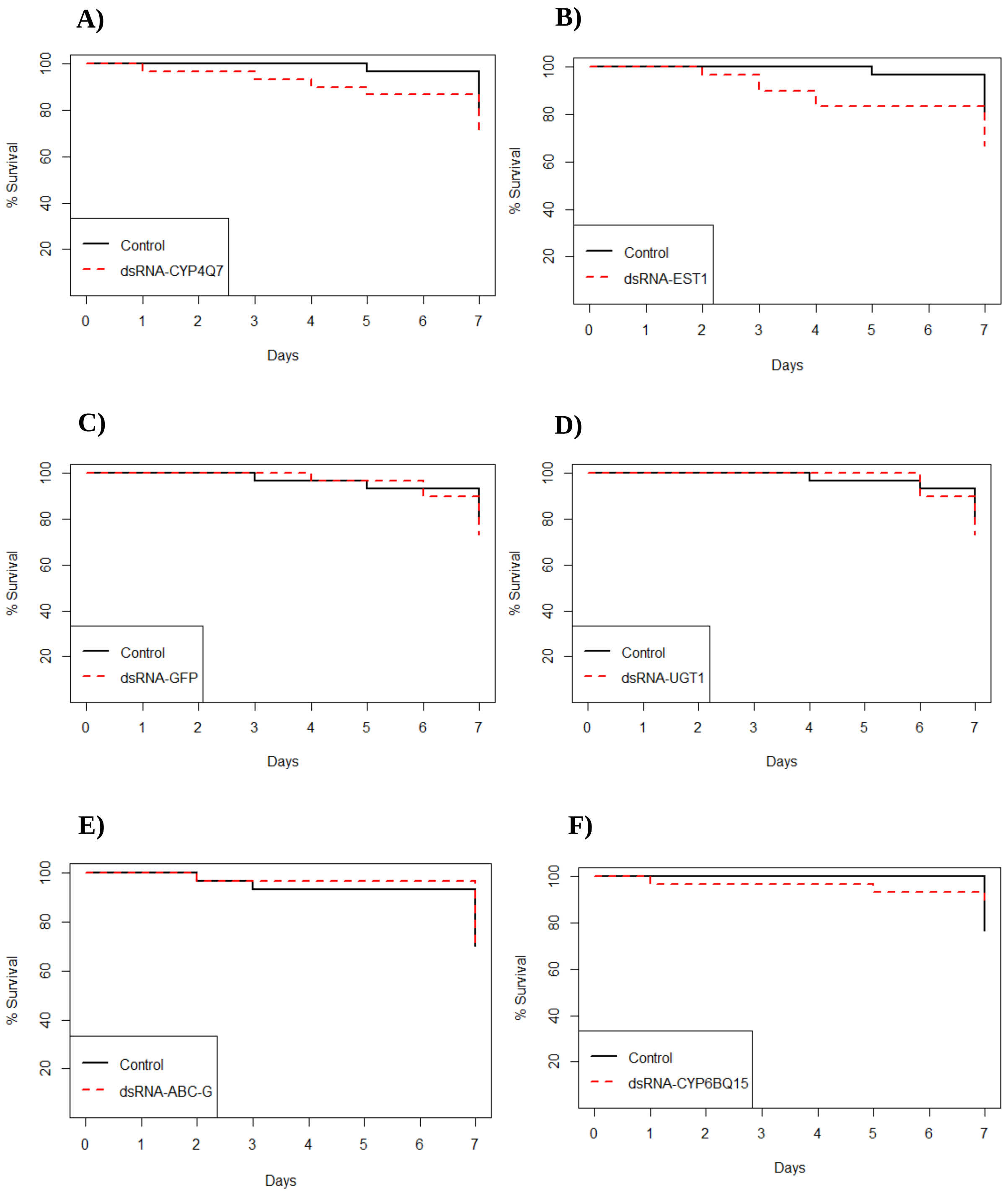


**Figure S3. Kaplan-Meier survival curves illustrating the percent survival of the RS beetles exposed to $LD_{20}$ of imidacloprid after RNAi of target genes.** Beetles either ingested *E. coli* HT115 (control) or *E. coli* HT115 producing dsRNA for **A)** *CYP4Q7*, **B)** *EST1*, **C)** *GFP*, **D)** *UGT1*, **E)** *ABC-G*, and **F)** *CYP6BQ15* genes.

**Table S1. Summary of mRNA-seq data before and after mapping.**

| Sample name | Total raw reads | Mapped reads | % mapped | Uniquely mapped | % uniquely mapped |
|---|---|---|---|---|---|
| **RS biorep1** | 58,892,932 | 51,250,746 | 87.0 | 22,586,506 | 44.1 |
| **RS biorep2** | 62,045,496 | 53,781,131 | 86.7 | 22,718,902 | 42.2 |
| **RS biorep3** | 55,059,038 | 48,065489 | 87.3 | 20,613,349 | 42.9 |
| **SS biorep1** | 59,130,951 | 51,889,523 | 87.7 | 21,596,640 | 41.6 |
| **SS biorep2** | 61,911,228 | 53,098,114 | 85.8 | 22,677,904 | 42.7 |
| **SS biorep3** | 53,963,077 | 46,221,323 | 85.6 | 20,124,899 | 43.5 |

**Table S2. List of significantly differentially expressed contigs encoding detoxifying enzymes (CYP, EST, UGT, and GST) and ABC transporters in the RS beetles compared to the SS beetles.**

| **Contig ID[1]** | **Sequence Description[2]** | **Read count SS[3]** | **Read count RS[3]** | **Log2 Fold change** | ***P*-adj[4]** | **Regulation In RS** |
|---|---|---|---|---|---|---|
| **CYPs** | | | | | | |
| **Ld_rep_c34031** | ***CYP6BQ15*** | **8.33** | **943.26** | **6.82** | **1.04E-32** | **Up** |
| Ld_rep_c51084 | *CYP6K1* | 0.32 | 25.86 | 6.32 | 1.21E-09 | Up |
| Ld_c20712 | *CYP4G57* | 1.61 | 108.33 | 6.07 | 8.18E-28 | Up |
| Ld_rep_c41850 | *CYP6BJ1* | 0.65 | 40.4 | 5.97 | 8.94E-05 | Up |
| Ld_rep_c61559 | *CYP6EF1* | 0.33 | 16.29 | 5.61 | 7.99E-06 | Up |
| Ld_rep_c91876 | *CYP9Z12V1* | 0.32 | 10.78 | 5.06 | 7.62E-04 | Up |
| **Ld_rep_c33314** | ***CYP4Q3*** | **61.04** | **960.36** | **3.98** | **2.77E-38** | **Up** |
| Ld_rep_c48733 | predicted *CYP* | 5.07 | 73.43 | 3.86 | 1.45E-12 | Up |
| Ld_rep_c25417 | *CYP4Q* | 113.41 | 1592.4 | 3.81 | 7.08E-22 | Up |
| Ld_rep_c36308 | *CYP6BU1* | 2.92 | 34.48 | 3.56 | 6.57E-08 | Up |
| Ld_rep_c27085 | *CYP412A2* | 31.1 | 210.53 | 2.76 | 1.40E-15 | Up |
| Ld_c756 | *CYP412A2* | 59.35 | 354.61 | 2.58 | 3.02E-16 | Up |
| Ld_rep_c45335 | *CYP9Z14V3* | 14.79 | 81.61 | 2.46 | 1.59E-05 | Up |
| Ld_rep_c63019 | *CYP413A1* | 13.15 | 67.8 | 2.37 | 1.48E-07 | Up |
| Ld_c981 | *CYP12A4* | 504.67 | 2435.68 | 2.27 | 2.83E-16 | Up |
| Ld_c259 | *CYP6BQ15* | 1995.9 | 7776.35 | 1.96 | 5.78E-06 | Up |
| Ld_rep_c30474 | *CYP301B1* | 62.9 | 240.41 | 1.93 | 4.53E-06 | Up |
| Ld_c20506 | *CYP6EH1* | 823.72 | 3049.34 | 1.89 | 2.55E-04 | Up |
| Ld_c72702 | *CYP12H2* | 261.14 | 910.62 | 1.8 | 3.68E-10 | Up |
| Ld_rep_c75503 | *CYP6BQ16* | 104.44 | 341.18 | 1.71 | 3.90E-04 | Up |
| Ld_rep_c24490 | *CYP6BQ15* | 802.12 | 2476.07 | 1.63 | 4.88E-09 | Up |
| Ld_c22309 | *CYP314A1* | 161.76 | 445.14 | 1.46 | 7.85E-06 | Up |
| Ld_c55986 | *CYP314A1* | 240.19 | 617.71 | 1.36 | 5.60E-05 | Up |
| Ld_rep_c68743 | *CYP4G57* | 0 | 228.6 | NA | 9.88E-34 | Up |
| Ld_rep_c34317 | *CYP6BQ15* | 145.65 | 30.33 | -2.26 | 3.65E-10 | Down |

| | | | | | | |
|---|---|---|---|---|---|---|
| Ld_rep_c75371 | *CYP412A1* | 52.29 | 9.77 | -2.42 | 1.29E-06 | Down |
| Ld_c20095 | *CYP412A2* | 41.63 | 7.6 | -2.45 | 1.15E-05 | Down |
| **Ld_rep_c34168** | ***CYP4Q7*** | **100.01** | **15.34** | **-2.71** | **4.76E-11** | **Down** |
| Ld_rep_c60423 | *CYP4C1* | 107.94 | 2.39 | -5.5 | 2.42E-19 | Down |
| Ld_rep_c48659 | *CYP6BK17* | 43.89 | 0.66 | -6.06 | 2.11E-14 | Down |
| **ESTs** | | | | | | |
| Ld_rep_c71421 | *EST4* | 0.31 | 194.83 | 9.28 | 3.74E-32 | Up |
| Ld_rep_c36657 | *Carboxyl EST1* | 0.32 | 82.38 | 7.99 | 8.41E-19 | Up |
| Ld_rep_c34698 | *EST5* | 3.53 | 880.32 | 7.96 | 2.97E-31 | Up |
| Ld_rep_c77075 | *EST Beta* | 0.32 | 51.99 | 7.33 | 2.29E-05 | Up |
| Ld_rep_c35289 | *Acetlycholin EST1* | 5.15 | 292.39 | 5.83 | 7.48E-42 | Up |
| Ld_rep_c53802 | *EST FE4* | 1.92 | 65.66 | 5.1 | 5.85E-16 | Up |
| Ld_rep_c35399 | *EST2* | 14.51 | 389.18 | 4.75 | 1.44E-22 | Up |
| Ld_rep_c46562 | *EST3* | 5.26 | 121.55 | 4.53 | 5.34E-15 | Up |
| **Ld_c2942** | ***EST1*** | **299.34** | **4078.42** | **3.77** | **7.68E-39** | **Up** |
| Ld_rep_c24217 | *EST6* | 730.27 | 2934.8 | 2.01 | 3.25E-10 | Up |
| Ld_c2931 | *Carboxyl EST2* | 134.3 | 530.97 | 1.98 | 2.49E-09 | Up |
| Ld_rep_c25830 | *Acetyl Cholinest2* | 1409.01 | 3374.43 | 1.26 | 5.83E-04 | Up |
| Ld_c5150 | *EST7* | 1084.67 | 2203.66 | 1.02 | 7.14E-04 | Up |
| Ld_rep_c28597 | *EST8* | 0 | 181.41 | NA | 5.25E-46 | Up |
| Ld_rep_c36550 | *EST FE4* | 60.88 | 0 | NA | 1.27E-21 | Down |
| Ld_rep_c34853 | *Venom Carboxyl EST6* | 73.02 | 0 | NA | 7.20E-25 | Down |
| Ld_rep_c68979 | *Carboxyl EST4* | 373.43 | 107.2 | -1.8 | 5.46E-06 | Down |
| Ld_rep_c33690 | *EST10* | 245.23 | 47.19 | -2.38 | 1.58E-11 | Down |
| Ld_rep_c33908 | *EST FE4* | 353.71 | 51.71 | -2.77 | 6.86E-09 | Down |
| Ld_rep_c36417 | *EST FE4* | 528.18 | 74.76 | -2.82 | 2.35E-09 | Down |
| Ld_rep_c24505 | *Acetyl Cholinest* | 1924.85 | 185.92 | -3.37 | 7.24E-06 | Down |
| Ld_rep_c24395 | *EST11* | 47.12 | 1.01 | -5.55 | 1.59E-14 | Down |
| Ld_rep_c26610 | *Alpha EST* | 469.03 | 1.73 | -8.08 | 4.67E-21 | Down |

| | | | | | | |
|---|---|---|---|---|---|---|
| **GSTs** | | | | | | |
| Ld_rep_c33018 | *GST Sigma1* | 3.24 | 2034.78 | 9.3 | 1.00E-106 | Up |
| Ld_rep_c40253 | *GST Sigma2* | 1.58 | 91.86 | 5.86 | 1.99E-13 | Up |
| Ld_rep_c24170 | *GST1* | 38.51 | 2058.82 | 5.74 | 1.88E-68 | Up |
| Ld_rep_c41971 | *GST Sigma3* | 4.2 | 105.26 | 4.65 | 2.79E-22 | Up |
| Ld_rep_c24256 | *GST Delta1* | 232.16 | 1275.78 | 2.46 | 4.29E-18 | Up |
| Ld_rep_c26032 | *GST Epsilon6* | 96.75 | 449.15 | 2.21 | 2.92E-13 | Up |
| Ld_rep_c44006 | *GST2* | 27.08 | 122.07 | 2.17 | 6.89E-09 | Up |
| Ld_c19072 | *GST2C1-Like* | 12.51 | 50.78 | 2.02 | 4.86E-05 | Up |
| Ld_rep_c24751 | *GST Sigma4* | 2715.74 | 7397.42 | 1.45 | 1.02E-04 | Up |
| Ld_rep_c38387 | *GST Epsilon* | 574.55 | 1438.38 | 1.32 | 5.21E-06 | Up |
| Ld_rep_c33334 | *GST Omega1* | 389.81 | 892.55 | 1.2 | 6.62E-05 | Up |
| Ld_rep_c46479 | *GST Theta* | 158.96 | 359.43 | 1.18 | 2.62E-04 | Up |
| Ld_rep_c25066 | *GST Epsilon3* | 799.76 | 1757.33 | 1.14 | 1.25E-04 | Up |
| Ld_rep_c50771 | *GST3* | 0 | 36.27 | NA | 1.38E-04 | Up |
| Ld_rep_c48065 | *GST Delta2* | 0 | 107.98 | NA | 2.25E-33 | Up |
| Ld_rep_c54053 | *GST Epsilon7* | 474.9 | 171.67 | -1.47 | 1.18E-06 | Down |
| Ld_rep_c43735 | *GST5* | 939.47 | 148.33 | -2.66 | 2.79E-20 | Down |
| Ld_rep_c34301 | *GST* | 249.18 | 38.28 | -2.7 | 2.72E-13 | Down |
| Ld_rep_c38198 | *GST7* | 815.64 | 0.69 | -10.2 | 7.68E-86 | Down |
| **UGTs** | | | | | | |
| Ld_rep_c84840 | *UGT2C1* | 0.33 | 356.14 | 10.06 | 1.31E-52 | Up |
| **Ld_rep_c41594** | ***UGT1*** | **0.65** | **192.9** | **8.22** | **7.10E-30** | **Up** |
| Ld_rep_c83152 | *UGT7* | 0.64 | 124.34 | 7.61 | 5.06E-20 | Up |
| Ld_rep_c45975 | *Antennal enriched UGT* | 2.61 | 70.11 | 4.75 | 1.46E-17 | Up |
| Ld_rep_c28339 | *UGT2B15* | 23.96 | 95.58 | 2 | 6.09E-07 | Up |
| Ld_rep_c58571 | *UGT3* | 16.15 | 63.67 | 1.98 | 1.37E-05 | Up |
| **Ld_c190** | ***UGT2*** | **147.93** | **522.5** | **1.82** | **9.03E-10** | **Up** |
| Ld_c269 | *UGT4* | 409.05 | 1174.08 | 1.52 | 3.64E-04 | Up |
| Ld_rep_c39043 | *UGT2B23* | 54.94 | 144.31 | 1.39 | 1.23E-04 | Up |
| Ld_rep_c33389 | *UGT5* | 576.6 | 1281.78 | 1.15 | 1.08E-04 | Up |

| | | | | | | |
|---|---|---|---|---|---|---|
| Ld_rep_c38005 | *Antennal-enriched UGT* | 0 | 143.76 | NA | 1.18E-34 | Up |
| Ld_rep_c35232 | *UGT6* | 0 | 56.36 | NA | 1.96E-10 | Up |
| Ld_rep_c30928 | *UGT2C1* | 0 | 458.59 | NA | 1.77E-72 | Up |
| Ld_rep_c28388 | UGT7 | 543.8 | 0.35 | -10.62 | 5.63E-75 | Down |
| Ld_rep_c84951 | *UGT2C1-like* | 71.06 | 21.57 | -1.72 | 8.60E-05 | Down |
| **ABC transporters** | | | | | | |
| **Ld_rep_c28427** | ***ABC-G*** | **0.66** | **22.29** | **5.09** | **5.20E-05** | **Up** |
| Ld_rep_c27116 | *MRP 4-1* | 0.66 | 12.63 | 4.27 | 4.95E-04 | Up |
| Ld_c11003 | *ABC-B6* | 3.88 | 61.31 | 3.98 | 1.53E-07 | Up |
| Ld_c571 | *MRP 4-2* | 37.77 | 285.89 | 2.92 | 5.86E-08 | Up |
| Ld_rep_c26545 | *ABC-B6 mitochondrial* | 241.68 | 615.56 | 1.35 | 6.66E-06 | Up |
| Ld_c62808 | *MRP-2* | 0 | 80.57 | NA | 2.90E-27 | Up |
| Ld_rep_c34742 | *MRP-4-3* | 0 | 291.03 | NA | 4.25E-59 | Up |
| Ld_c24118 | *MRP-4 like4* | 0 | 14.12 | NA | 5.25E-06 | Up |
| Ld_c7947 | *MRP like* | 123.94 | 47.61 | -1.38 | 2.40E-04 | Down |
| Ld_c12043 | *MRP 4-like5* | 275.31 | 91.46 | -1.59 | 7.75E-07 | Down |
| Ld_c73069 | *MRP like* | 399.13 | 121.75 | -1.71 | 2.47E-08 | Down |
| Ld_c24114 | *MRP 4-like 5* | 416.18 | 117.69 | -1.82 | 2.03E-09 | Down |
| Ld_c56678 | *MRP* | 334.54 | 38.75 | -3.11 | 3.47E-07 | Down |
| Ld_c6433 | *MRP-1* | 368.52 | 11.59 | -4.99 | 2.73E-41 | Down |
| Ld_rep_c91275 | *MRP 4-4* | 619.43 | 0.35 | -10.81 | 6.23E-79 | Down |

[1]Contig ID from the reference transcriptome[33]

[2]Genes selected for qPCR validation of mRNA-seq data are bolded

[3]Read counts represent mean normalized counts from three biological replicates

[4]Adjusted *P*-value based on false discovery rate (FDR)[55]-corrected α cut-off of 0.001

NA = not available

**Table S3. Estimated fold change differences for seven genes in the RS beetles compared to the SS beetles from qPCR and DESeq analyses.**

| **Contig ID**[1] | **Gene**[2] | **Fold change in qPCR**[3] | **Fold change in DESeq** | **Trend**[4] |
|---|---|---|---|---|
| **Ld_rep_c33314** | ***CYP4Q3*** | 5.79 | 15.73 | S |
| **Ld_rep_c34031** | ***CYP6BQ15*** | 79.38 | 113.21 | S |
| **Ld_rep_c34168** | ***CYP4Q7*** | 7.24 | 0.15 | O |
| **Ld_c2942** | ***EST1*** | 6.22 | 13.62 | S |
| **Ld_c190** | ***UGT2*** | 3.91 | 3.53 | S |
| **Ld_rep_c41594** | ***UGT1*** | 1565.56 | 298.79 | S |
| **Ld_rep_c28427** | ***ABC-G*** | 35.67 | 33.98 | S |

[1]Contig ID from the reference transcriptome[33]

[2]Genes selected for RNAi studies are bolded

[3]qPCR fold changes are means from independent biological samples

[4]S = same trend and O = opposite trend in estimated transcript levels of the genes from two methods

**Table S4. Nucleic acid sequences used for production of dsRNA in *E. coli* HT115.**

| Contig ID[1] | Sequence Description | Sequence[2] |
|---|---|---|
| **Ld_rep_c34031** | *CYP6BQ15* | AACATCCTCACGGACCATTCCATTATTGGCGATGC<br>CATAGACATCAAGGATGTCGTATCCCGATTCACAA<br>CTGACGTGATAGGTTCTGTAGCTTTCGGAATAGAY<br>TGTAATAGTCTCAAGGATCCCGACTCCGAATTCAG<br>GCATTGGGGAAAAAGGATATTCACGTTCGATTTTA<br>TGAGGAGAATCAAGAACAATATAACAATGTTGATT<br>CCGAGGGATATTGTGATCAAGACAGGCATAAAATT<br>GATGTCACGTGACTTGGAAGACTTCTTCATGAACG<br>TGGTCAGAAGCACAGTTCAGTTCAGAGAAACTCAC<br>AACGTCCATAGGAAGGATTTCATGCACTTGCTGTT<br>ACAACTGAAGAATAAAGGACAAATTGCTGAGGAC<br>GATAGCACTGATAAAGAAATCGAAATTAAGGCAC<br>CC |
| **Ld_rep_c34168** | *CYP4Q7* | CATCTCCTGACGTCCGAATCCACTAAAATTGTAGT<br>AAATACATGAGATGTATAGCCATGGTTTCTTCAAC<br>CTCTGGARAAATATGTGTCCCATTTTATTAATGGAA<br>GTTATGTATTCTTTATCCTTTTTAGTTTTCTGGTTCA<br>GTTTGGTCCCCATGGATGATTCTGCGATAGTATTCA<br>ATGTAAACTCTGATATCAATGGAAAGACATTGGTC<br>GAGGTTTTTTCAGTTTCATTTCTAAGAACTTCCACT<br>AATTTTTTCGTTTCATTATTGAAAACTCCAACAAAT<br>TGCTGCAAAATACTGRAATGAAAGGCTGGTGTCAM<br>GATTTTTCGACGAGTCTGCCATTTGGAACCTGTACT<br>TGTCAACAAGCCTTCTCCTAGCCAGCGATTCA |
| **Ld_rep_c33314** | *CYP4Q3* | TTGGACCAGCAATCGCCTCAAATTCCTCTGCACCT<br>GTAATGTTCACACCAGGGTAGAGAGAAGTCATGTC<br>AATTCTGTAAATTGGTCCGTATGTTCTAGCCCAGTA<br>TCTCACCTTGCGGAATAACGTTATATCGTCGCCCA<br>AAAATTCAAGAACATTTTTCAAGACGGGTATAGGT<br>TTTGGCCCAGGCAGTCCTTTCAAAATTTTCAACGTT<br>CTCAAATGCTCGATGAAACCTCTCACAAGAAAAAA<br>TAATATCACACATCCTGCCACCACAACTGAAAAAT<br>TTACTATAAACATTTCAGTATATTCAATTCAAAAGT<br>TCTTTGTCAAGAACAATCGTCTGAATAAATCTACT<br>ACGCGTTGTGAACGCTGTCAATAATTTTTCTATTCG<br>AACAACTTTTGAAGTGTTTTCGTGCGA |

| | | |
|---|---|---|
| **Ld_c2942** | *EST1* | TCGAATCCAACAAGTGGTGATCCACTTTCCATGAT<br>AGCTCCACGGAAGAGCTCTTCTCCTCCATTTTTCTG<br>TGCTAATACATGCAGACTCACAGATAATGAACCTG<br>AACTTTCACCTACAAGGGTAACTTTTGCAGGATCT<br>CCTCCAAATAAATGAATATTTTCATTCACCCATTTA<br>ATTGCAAACTTTTGATCTTTCAGGCCGATGTTGGCC<br>GGTATAATGTCATCTTCTGTTGTGGTGAATCCAAAT<br>GGCCCCAATCGGTAATTGAAAGTTACTACAATTAT<br>CTCATGATCCATGATGTATTTTGGTCCAGAATATTC<br>ATATGTACTTGATTCTGATGATAAACCTCCACCATG<br>TATCCAAAGTAAAACTGGCAATTTCTTGGATTTACT<br>ACCAGGTTTCAGCGGC |
| **Ld_rep_c28427** | *ABC-G* | TGGTGACTTTTCCACTGGGAATCGATTCTTTGTCTT<br>GTGGGGTCAACATGTCTCCCCTCGTTACACTGAGT<br>CTCCCCTCATCTTGCTCTTTACTGAGCATCAGAAAA<br>ACGTCTTCTAATGTTTGACTATTGTAAAAAGCCAA<br>AAGCCTTTCCGGGTTCTCTTGAGCCAAAATGATTCC<br>CCCTCTCAACAAACAGATCTTATCAGTCTGCCTAC<br>ATTCCTCAATATAGTGTGTGGTTATTATCACAGAA<br>GTTCCTAGTTTCTTCGTCATATCGACTAGATAGTTC<br>CATATTCTCTCCCTCAAAAGTGGATCAACTCCCACC<br>GTTGGTTCATCCAGTATTAACAACTCTGGTTTGTGG<br>ATAACTGCCGAGGCAAAGGACACTCTTC |
| **Ld_rep_c41594** | *UGT1* | TCACTCATGGCGGTTTGTTGAGTACAACAGAAACA<br>ATCTATCATGGTGTACCAATACTCGCTATTCCTGTT<br>TTTGGGGATCAGAAGATGAATGCTGCAAAGGCGGT<br>TGCAGCAGGATATGGTTTATCTTTATCAATAAACG<br>AGTTGAGCGAGGAAAACTTATCTAACAGCATAAAT<br>GAACTTTTGAATAATTCGAAGTATAGGGACAATGC<br>AAAAAGAAGATCCGCAATCATGCACGATCGAAAA<br>GTGAAGCCCATGGATCTTGCAACATATTGGATCGA<br>ATTTGTAGTCCGACATAAAGGAGCACCACATTTGA<br>GGGTAGCTGCCCTTGATTTAACTTGGTACCAATATT<br>TCCTCGTGGATATTGTTCTTCTTGTGGGAGCTGTTG<br>TTGCCAGTTTGATGCTAGCGTC |
| **Ld_c190** | *UGT2* | TTTCCATCTCCGCATGAAATATTGCTGAAACTTGGA<br>GAACAACTTATTTATAATGTAGCAGAATAAGAATG<br>AAGTGAAGAATATAAATCCCAAAATATCCAACAA |

| | | |
|---|---|---|
| | | GTAAAACTGGTAGAGCGGTATGTCTGAAGCGGGGT<br>TTCGTAGCTCTTTGGCTCCTCTATTCCTTATAACGT<br>ATTCAGTCCACCATACAGCCTTTTCCAAACCACTCA<br>TTGGTTCATCACCAAGAAGACTGGCAAGCTTTCTA<br>ATGGACGATTTATACTTCTCATTATGTGTAACTTCC<br>ATGATGGCATCTCTCAAATCCTTATAGCTCAAAGC<br>CGGTTTATGATAAATTTGTTTCCCAATATTTTTATT<br>CTCTACGATTCGAGCGTTTTTCAGCTGATCTCCGAA<br>GAACGGCATAGCTA |
| - | *GFP* | CCATGCCCGAAGGTTATGTACAGGAAAGAACTATA<br>TTTTTCAAAGATGACGGGAACTACAAGACACGTAA<br>GTTTAAACAGTTCGGTACTAACTAACCATACATAT<br>TTAAATTTTCAGGTGCTGAAGTCAAGTTTGAAGGT<br>GATACCCTTGTTAATAGAATCGAGTTAAAAGGTAT<br>TGATTTTAAAGAAGATGGAAACATTCTTGGACACA<br>AATTGGAATACAACTATAACTCACACAATGTATAC<br>ATCATGGCAGACAAACAAAAGAATGGAATCAAAG<br>TTGTAAGTTTAAACATGATTTTACTAACTAACTAAT<br>CTGATTTAAATTTTCAGAACTTCAAAATTAGACAC<br>AACATTGAAGATGGAAGCATTCAACTAGCAGACCA<br>TTATCAACAAAATACTCCAATTGGCGATGGCCCTG<br>TCCTTTTACCAGACAACCATTACCTGTCC |

[1]Contig ID from reference transcriptome[33]

[2]Primers used (without restriction enzyme cut sites) are underlined

**Table S5. Topical bioassay results data used to calculate resistance ratio at $LD_{50}$ of imidacloprid in adult CPB**

| CPB Strain | Dose (µg) | BR | N | Time period (days) | | | | | | | | | | | | | | Total (M+D) | % mortality[1] | % corrected mortality[2] | Approximate $LD_{50}$ RR[3] |
|---|---|---|---|---|---|---|---|---|---|---|---|---|---|---|---|---|---|---|---|---|---|
| | | | | 1 | | 2 | | 3 | | 4 | | 5 | | 6 | | 7 | | | | | |
| | | | | M | D | M | D | M | D | M | D | M | D | M | D | M | D | | | | |
| SS | 0.0 (acetone control) | 1 | 10 | 0 | 0 | 0 | 0 | 0 | 0 | 0 | 0 | 0 | 0 | 0 | 0 | 0 | 0 | 0 | 0 | 53 | 25.3 |
| | | 2 | 10 | 0 | 0 | 0 | 0 | 0 | 0 | 0 | 0 | 0 | 0 | 0 | 0 | 0 | 0 | 0 | | | |
| | | 3 | 10 | 2 | 0 | 0 | 0 | 0 | 0 | 0 | 0 | 0 | 0 | 0 | 0 | 0 | 0 | 0 | | | |
| | 0.19 | 1 | 10 | 8 | 0 | 5 | 1 | 3 | 2 | 4 | 2 | 4 | 2 | 3 | 2 | 1 | 3 | 4 | 53 | | |
| | | 2 | 10 | 9 | 0 | 8 | 0 | 5 | 0 | 5 | 0 | 7 | 0 | 4 | 2 | 3 | 3 | 6 | | | |
| | | 3 | 10 | 7 | 1 | 3 | 1 | 6 | 2 | 4 | 2 | 5 | 2 | 5 | 2 | 4 | 2 | 6 | | | |
| RS | 0.0 (acetone control) | 1 | 10 | 2 | 0 | 0 | 0 | 0 | 0 | 0 | 0 | 0 | 0 | 0 | 0 | 0 | 0 | 0 | 3.33 | 55 | |
| | | 2 | 10 | 0 | 0 | 0 | 0 | 0 | 0 | 0 | 0 | 0 | 0 | 0 | 0 | 0 | 0 | 0 | | | |
| | | 3 | 10 | 0 | 0 | 0 | 0 | 0 | 0 | 0 | 1 | 0 | 1 | 0 | 1 | 0 | 1 | 1 | | | |
| | 4.8 | 1 | 10 | 10 | 0 | 9 | 0 | 6 | 1 | 8 | 1 | 6 | 2 | 3 | 3 | 5 | 1 | 6 | 56.67 | | |
| | | 2 | 10 | 10 | 0 | 7 | 0 | 5 | 0 | 8 | 0 | 3 | 2 | 2 | 3 | 2 | 3 | 5 | | | |
| | | 3 | 10 | 7 | 1 | 4 | 1 | 4 | 1 | 5 | 1 | 3 | 2 | 3 | 2 | 4 | 2 | 6 | | | |

**M**= total number of moribund beetles at given day

**D**= total number of dead beetles at a given day

**BR** = biological replicate

**N**= number of adult beetles used per biological replicate

[1]Percent mortality was calculated by dividing total number of dead and moribund beetles at the end of 7th day by total number of beetles used in the bioassay

[2]Percent corrected mortality was calculated using Abbott's formula

[3]Approximate $LD_{50}$ resistance ratio (RR) was calculated by dividing approximate $LD_{50}$ of imidacloprid for RS by approximate $LD_{50}$ of imidacloprid for SS

**Table S6. List of primers used in qPCR analysis.**

| Contig ID[1] | Gene | Forward and reverse primers (5'-3') | Primer Efficiency (%)[2] | Amplicon size (bp)[3] |
|---|---|---|---|---|
| **Ld_rep_c33314** | *CYP4Q3* | TACCCTGGTGTGAACATTAC<br>AATGAAAGGCTGGTGTCAAG | 97.1 | 178 |
| **Ld_rep_c34031** | *CYP6BQ 15* | TAGGCTGACCCCAACATTCA<br>AATGGAATGGTCCGTGAGGA | 93.4 | 103 |
| **Ld_rep_c34168** | *CYP4Q7* | CAGCCTAAGACTTCCTTGATG<br>GTTCGAGGGATTTGACACTAC | 96.1 | 193 |
| **Ld_rep_c28427** | *ABC-G* | TCACCTCCACTACAGTCAAC<br>GCTCTGGTGGAAAGTCTAAC | 99.6 | 158 |
| **Ld_rep_c41594** | *UGT1* | AGCACCACATTTGAGGGTAG<br>GGTGAGTGAAGATGAGATCC | 95.9 | 207 |
| **Ld_c190** | *UGT2* | TCTCCGAAGAACGGCATAG<br>GAGTCATCTCTTCCCTTGAATGT | 95.2 | 176 |
| **Ld_c2942** | *EST1* | ACCCTGCCACTTTTCCACTT<br>ACTGACACAATCGGTGACG | 94.4 | 177 |
| **-** | *L8E* | GGTAACCATCAACACATTGG<br>TCTTGGCATCCACTTTACC | 97.4 | 124 |
| **-** | *ARF1* | GACTGCAAGTAGGAGAAGTTG<br>TCGGCAGAGTCTACCACAT | 94.1 | 181 |
| **-** | *EF1A* | CAGGGCAAGGTTTGAAAGATAA<br>CCATCAGCACAGTTCCCAT | 99.6 | 168 |

[1]Contig ID from reference transcriptome[33]

[2]Primer efficiencies were tested by generating standard curves following the guidelines described[59]

[3]PCR products were sequenced to confirm amplification of correct sequences

**Table S7. List of primers used in cloning and sequencing of plasmid constructs.**

| Contig ID[1] | Gene | Forward and reverse primers (5'-3')[2] | Product size (bp) |
|---|---|---|---|
| **Ld_rep_c34031** | *CYP6BQ15* | TAGCGGCCGCAACATCCTCACGGACCATTC<br>ACAGGTCGACGGGTGCCTTAATTTCGATTTC | 420 |
| **Ld_rep_c34168** | *CYP4Q7* | TAGCGGCCGCCATCTCCTGACGTCCGAATC<br>ACAGGTCGACTGAATCGCTGGCTAGGAGAAG | 387 |
| **Ld_rep_c33314** | *CYP4Q3* | TAGCGGCCGCTTGGACCAGCAATCGCCT<br>ACAGGTCGACTCGCACGAAAACACTTCAAA | 413 |
| **Ld_c2942** | *EST1* | TAGCGGCCGCTCGAATCCAACAAGTGGTGA<br>ACAGGTCGACGCCGCTGAAACCTGGTAGTA | 408 |
| **Ld_rep_c28427** | *ABC-G* | TAGCGGCCGCTGGTGACTTTTCCACTGGG<br>ACAGGTCGACGAAGAGTGTCCTTTGCCTC | 384 |
| **Ld_rep_c41594** | *UGT1* | TAGCGGCCGCTCACTCATGGCGGTTTGTTG<br>ACAGGTCGACGACGCTAGCATCAAACTGGC | 409 |
| **Ld_c190** | *UGT2* | TAGCGGCCGCTTTCCATCTCCGCATGAAAT<br>ACAGGTCGACTAGCTATGCCGTTCTTCG | 404 |
| - | *GFP* | TAGCGGCCGCCCATGCCCGAAGGTTATGTA<br>ACAGGTCGACGGACAGGTAATGGTTGTCTGG | 449 |
| - | L4440 sequencing primers | GACCGGCAGATCTGATATCATC<br>CTCACTGGCCGTCGTTTTAC | - |

[1]Contig ID from the reference transcriptome[33]

[2] *Not*I and *Sal*I restriction enzyme cut sites are underlined

**Table S8. Plasmid constructs used in this study.**

| Plasmid[1] | Description | Source |
|---|---|---|
| **L4440, $Amp^R$** | RNAi feeding vector, empty backbone | Addgene plasmid # 1654 |
| **GFP::L4440, $Amp^R$** | Contains full length *GFP* sequence | Addgene plasmid # 11335 |
| **GFP-RNAi::L4440, $Amp^R$** | dsRNA production for *GFP* control | This study |
| **CYP4Q3::L4440, $Amp^R$** | dsRNA production for *CYP4Q3* | This study |
| **CYP4Q7::L4440, $Amp^R$** | dsRNA production for *CYP4Q7* | This study |
| **CYP6BQ15::L4440, $Amp^R$** | dsRNA production for *CYP6BQ15* | This study |
| **ABC-G::L4440, $Amp^R$** | dsRNA production for *ABC-G* | This study |
| **UGT1::L4440, $Amp^R$** | dsRNA production for *UGT1* | This study |
| **UGT2::L4440, $Amp^R$** | dsRNA production for *UGT2* | This study |
| **EST1::L4440, $Amp^R$** | dsRNA production for *EST1* | This study |

[1]$Amp^R$ is resistance to ampicillin (β-lactamase)
